# Editorial: Horticultural crops: Interaction with fungal community

**DOI:** 10.3389/fpls.2022.1096201

**Published:** 2022-12-06

**Authors:** Zhongwei Zou, Xujun Zhu, Sanjaya Gyawali

**Affiliations:** ^1^ Department of Biology, Wilfrid Laurier University, Waterloo, ON, Canada; ^2^ College of Horticulture, Nanjing Agricultural University, Nanjing, Jiangsu, China; ^3^ Sakata Seed America Inc, PNW Plant Pathology Laboratory, Burlington, WA, United States

**Keywords:** horticultural crop, fungal community, beneficial microbes, microbe-plant interaction, plant disease

Horticultural crops such as vegetables, fruits, or ornamentals are used to increase the diversity of diet nutrient, improve health, and beautify our living environment. They are prone to interact with various fungi and other microbes during multiple developmental stages of growth. These fungal pathogens cause significant yield and economical losses in horticultural crops annually. For example, fungal pathogens along with the mechanical wounds and environmental factors cause up to 90% of total post harvest losses in citrus species (Perez et al., 2017; Zhu et al., 2017; Lin et al., 2019). Besides the pathogenic effect of fungi pathogens, beneficial plant-microbe interactions also play a role in horticultural crops. *Trichoderma* species, e,g., are widely used as bio-fertilizer and bio-fungicide (Kubheka and Ziena, 2022; Modrzewska et al., 2022). Plant-microbe interactions have been extensively studied in crops such as rice, wheat, corn and others in last two decades. However, there are knowledge gaps in the molecular mechanisms, integrated pest management, fungal genetics and biology, and beneficial microbes in horticultural crops. Thus, this research topic tried to update the recent knowledge and progress on the following questions: (1) how the beneficial microbes including fungi interact and promote horticultural crop growth; (2) how the fungal pathogens attack horticulture plants; (3) how to dig out more resistance from plants against fungal pathogens.

To address these questions, a research topic was initiated aiming to identify additional genetics resistance to pathogens, beneficial bio-agents, and to reveal the mechanisms between horticultural crops and microbe interactions. These findings will facilitate integrated pest management in horticultural crops. After rigorous peer review, we collected four research and one review articles covering horticultural crops as oilseed rape (*Brassica napus*), citrus, Panax ginseng (*Panax ginseng* C.A. Meyer), *Artemisia selengensis*, etc. We hope these research objectives on plant and microbe interactions in different plant species can benefit each other, since these studies applied various methodologies such as genome wide identification, gene expression analysis, strain growth assays, morphological and physiological characterisation. To help our potential audience navigate the research objectives, we briefly highlighted the main findings of the accepted articles as follows:

Microbial volatiles have beneficial effects promoting plant growth and improving the disease resistance. Jiang et al. reported two volatile organic compounds (VOCs) emitted from the fungus *Cladosporium halotolerans* NGPF1 that can stimulate plant growth in culture media and soil conditions. Two VOCs, 2-methyl-butanal and 3-methyl-butanal, were identified and suggested to promote plant growth based on a gene expression analysis poining to the involvement of auxin and expansion signaling regulation. This study provides significant implications on how to apply VOCs for plant growth promotion in agricultural practice. We also collected one paper submitted by Jiang et al., which reported a novel plant growth-promoting Rhizobacteria strain JI39 identified from *Arthrobacter nicotinovorans*. This strain can promote shoot and root growth in Panax ginseng, an important medicinal plant in eastern Asia. Through a field trial, they found that the fresh weight of 2-year-old ginseng significantly increased by 24.7% with the treatment of 10^8^ CFU/mL suspension of the bacteria. The JI39 strain could improve the soil urease, phosphatase, invertase and catalase activities to promote ginseng growth. JI39 can be applied as a potential microbial fertilizer to benefit the ginseng industry.

*Artemisia selengensis* Turcz is an important edible and medicinal vegetable, and yield and deterioration of quality are frequently affected by powdery mildew (PM) disease in both field and greenhouse cultivation. Guo et al. identified the *Golovinomyces artemisiae* pathogen, using morphological observation and molecular analysis, that caused the powdery mildew disease on *A. selengensis*. In this paper, they indicated that PM-infected plant leaves showed significantly lower chlorophyll fluorescence, antioxidant system responses, and callose/lignin contents. Physiologically, the malondialdehyde (MDA), superoxide anion, peroxidase (POD) activities increased, while superoxide dismutase (SOD), catalase (CAT), and ascorbic acid (AsA) contents decreased, suggesting that lignin and protective enzymes were the key factors playing a role against PM infection. In conclusion, PM results in damage to photosynthesis and causes the imbalance of antioxidant system in *A. selengensis*. Taking the advantage of published genome sequences in *Brassica* species, Wang et al. identified 35 heat shock proteins 90 (Hsp90s) in *Brassica napus*, one of the most important oil crops worldwide. Hsp90 is a small heat shock protein that is highly conserved in eukaryotic cells and acts as a molecular chaperone (Pearl and Prodromou, 2000). In this study, the molecular characteristics, phylogenetic relationships, evolution, protein structure, and *cis*-elements in the promoters, were bioinformatically analysed. Sclerotinia stem rot caused by *Sclerotinia sclerotiorum* is one of the most devasting diseases in oilseed rape. Among the 35 Hsp90s, nine were validated to play important roles in response to *S. sclerotiorum* infection by published transcriptome data and qPCR analysis, in which six and three were up and down regulated, respectively. Meanwhile, 14 Hsp90s were identified to be highly involved in salt stress response.

In this research topic, we also included a review article written by Bhatta, who systematically summarized and reviewed the recent scientific progress on alternative management approaches of post harvest diseases (green and white mold) caused by *Penicillium* spp. in Citrus. Bhatta puts effort in collecting 325 published research articles, reports, news, reviews, book chapters and comprehensively describes alternative strategies to manage the white and green mold diseases caused by *P. italicum* and *P. digitatum* in an eco- and environmentally friendly manner. This includes the antagonistic micro-organisms (yeast, bacteria, fungi), natural plant products, bio-fungicides, chitosan and chitosan-based citrus coating, heat treatment, ionizing and non-ionizing irradiation, food additives, and synthetic elicitors. It will become a valuable reference for further research on plant resistance and fruit-pathogen interaction, proposing more sustainable strategies in fruit disease management.

We hope that the knowledge from the above-mentioned contributions can facilitate further success for the identification of more beneficial bioagents, environment-friendly methods to control disease, and additionally, improving our understanding of plant-microbe interactions. Finally, we greatly appreciate the journal editors, peer reviewers, and authors for their efforts and time on these research topics. We hope that our readers can identify valuable information that will benefit their research programs.

## Author contributions

All authors listed have made a substantial, direct, and intellectual contribution to the writing of original draft, review and final edition, and approved it for publication.
